# Taurine: A Potential Ergogenic Aid for Preventing Muscle Damage and Protein Catabolism and Decreasing Oxidative Stress Produced by Endurance Exercise

**DOI:** 10.3389/fphys.2017.00710

**Published:** 2017-09-20

**Authors:** Flávia G. De Carvalho, Bryan S. M. Galan, Priscila C. Santos, Kelly Pritchett, Karina Pfrimer, Eduardo Ferriolli, Marcelo Papoti, Júlio S. Marchini, Ellen C. de Freitas

**Affiliations:** ^1^Postgraduate Program in Food and Nutrition, Faculty of Pharmaceutical Sciences, São Paulo State University Sao Paulo, Brazil; ^2^Department of Nutrition, Exercise and Health Sciences, Central Washington University Ellensburg, WA, United States; ^3^Department of Internal Medicine, Ribeirão Preto Medical School, University of São Paulo Ribeirão Preto, Brazil; ^4^School of Physical Education and Sports of Ribeirão Preto, University of São Paulo Ribeirão Preto, Brazil

**Keywords:** triathlon, taurine, chocolate milk and aerobic parameters

## Abstract

The aim of this study was to evaluate the effects of taurine and chocolate milk supplementation on oxidative stress and protein metabolism markers, and aerobic parameters in triathletes.

**Methods:** A double-blind, crossover study was conducted with 10 male triathletes, aged 30.9 ± 1.3 year, height 1.79 ± 0.01 m and body weight 77.45 ± 2.4 kg. Three grams of taurine and 400 ml of chocolate milk (TAUchoc), or a placebo (chocolate milk) (CHOC) was ingested post exercise for 8 weeks. Oxidative stress marker levels, and 24 h urinary nitrogen, creatinine, and urea excretion were measured before and after 8 weeks of training and supplementation with TAUchoc or CHOC. A maximal incremental running test on a treadmill was performed in order to evaluate aerobic parameters: V_max_, heart rate (HR) and rate of perceived exertion (RPE).

**Results:** TAUchoc treatment during the 8 weeks resulted in increased taurine plasma levels (PRE 201.32 ± 29.03 μmol/L and POST 234.36 ± 35.51 μmol/L, *p* = 0.01), decreased malondialdehyde levels (19.4%, *p* = 0.03) and urinary nitrogen excretion (−33%, *p* = 0.03), and promoted positive nitrogen balance (*p* = 0.01). There were no changes in reduced glutathione (TAUchoc PRE 0.72 ± 0.08 mmol/L and POST 0.83 ± 0.08 mmol/L; CHOC PRE 0.69 ± 0.08 mmol/L and POST 0.81 ± 0.06 mmol/L), vitamin E plasma levels (TAUchoc PRE 33.99 ± 2.52 μmol/L and 35.95 ± 2.80 μmol/L and CHOC PRE 31.48 ± 2.12 μmol/L and POST 33.77 ± 3.64 μmol/L), or aerobic parameters, which were obtained in the last phase of the maximal incremental running test (V_max_ TAUchoc PRE 13 ± 1.4 km/h and POST 13.22 ± 1.34 km/h; CHOC PRE 13.11 ± 2.34 km/h and POST 13.11 ± 2.72 km/h), the heart rate values were TAUchoc PRE 181.89 ± 24.18 bpm and POST 168.89 ± 46.56 bpm; CHOC PRE 181.56 ± 2.14 bpm and POST 179.78 ± 3.4 bpm, and the RPE were TAUchoc PRE 8.33 ± 2.4 AU and POST 9.1 ± 2.1 AU; CHOC PRE 8.11 ± 4.94 AU and POST 8.78 ± 2.78 AU).

**Conclusion:** Taurine supplementation did not improve aerobic parameters, but was effective in increasing taurine plasma levels and decreasing oxidative stress markers, which suggests that taurine may prevent oxidative stress in triathletes.

## Introduction

Triathlon is a high intensity sport associated with increased production of free radicals and oxidative stress, which may compromise an athlete's performance (Bentley et al., 2008). This increase occurs naturally and it is well established that low-to-moderate levels of oxidants play multiple regulatory roles in cells, such as cell signaling (Powers and Jackson, 2008). However, overproduction of free radicals may damage cellular components. Therefore, the use of antioxidant compounds post exercise aims to prevent oxidative stress without limiting free radical production (Powers and Jackson, 2008). In order to minimize the effects of exercise, researchers are examining nutrients that may help prevent oxidative damage while also contributing to an athlete's higher energy requirements. Taurine has been referred to as a potent antioxidant due to the presence of sulfonic acid, which promotes the conversion of highly cytotoxic substances including chloride and hypochlorous acid into relatively stable chloramine (Tappaz, 2004; Zhang et al., 2004). Additionally hypotaurine, a taurine precursor, can act as a hydroxyl radical (OH·) scavenger and inhibit lipid peroxidation, and therefore prevent iron (${\text{Fe}}_{2}^{+}$) self-oxidation (Tadolini et al., 1995).

Furthermore, taurine is proposed to generate several physiological effects including regulation of calcium homeostasis in both skeletal muscle and cardiac tissue (Huxtable, 1992; De Luca et al., 2015), increased muscle force (Schaffer et al., 2010), improved lipid metabolism (Murakami, 2015), and increased insulin sensitivity (Vettorazzi et al., 2014). In addition taurine may improve carbohydrate metabolism and favor glycogen resynthesis (Ribeiro et al., 2010). In addition, some researchers have observed an improved time to exhaustion in runners (Lee et al., 2003) and cyclists (Zhang et al., 2004; Rutherford et al., 2010) with taurine supplementation.

According to De Luca et al. (2015) taurine supplementation increases the amino acid levels in skeletal muscle, promotes greater force, and improves resistance and recovery. These actions are related to an increase in calcium binding protein (calsequestrin1), which maintains high quantities of calcium in the sarcoplasmic reticulum, promoting higher availability of calcium for muscle contraction. Taurine is important to keep excitation-contraction coupling and muscle performance; however, the mechanisms that elucidate how taurine affects human endurance performance and the appropriate doses are still unclear (Galloway et al., 2008; De Luca et al., 2015).

Long period intervention studies with human requires attention especially in the dose, since it have to be safe for the participants. Even if there were no negative effects described in the literature about using more than 3 g of taurine, our research group decided to use an amount that has been used in past studies and showed positive effects and that is higher than those studies (1 g by Geib et al., 1994; 1.66 g by Rutherford et al., 2010; 2 g by Ra et al., 2015). Moreover, others studies that used more than 3 g did acute interventions (6 g by Zhang et al., 2004; Ishikura et al., 2008).

Additionally chocolate milk has been found to be an effective post exercise recovery aid for athletes (Karp et al., 2006; Pritchett et al., 2009; Gilson et al., 2010), due to it ideal carbohydrate and protein content that is similar to over the counter recovery beverages. Thus, it has also been suggested to be effective for replenishing depleted glycogen in the muscles (Lunn et al., 2012), enhancing recovery after high intensity exercise when muscle cells are more sensitive to nutrient uptake (Pritchett et al., 2009), and may improve body composition (Ferguson-Stegall et al., 2011). In addition, chocolate milk tastes good and is generally acceptable for athletes (Karp et al., 2006; Pritchett et al., 2009).

The provision of specific nutrients, such as taurine, associated with a post exercise recovery beverage, such as chocolate milk, may be effective in preventing oxidative stress and promoting recovery. Therefore, this study has two purposes, (1) to examine the effectiveness of the association of taurine with low fat chocolate milk post exercise for an 8-week period on markers of oxidative stress and protein catabolism, and (2) to examine the efficacy of taurine combined with low fat chocolate milk post exercise on aerobic capacity in triathletes.

## Methods

### Subjects

Ten well-trained male, long distance triathletes (age = 30.9 ± 1.3 years., stature = 1.79 ± 0.01 m, mass = 77.45 ± 2.4 kg; mean ± SD) who competed in semiprofessional triathlons volunteered to take part in this study. Their training was focused on the Brazilian Ironman Championships. The participants were invited to visit the lab where all experimental procedures and associated risks and benefits were explained. Those who agreed to participate were asked to provide written consent. Approval for this study was granted by the Human Ethics Committee of the State University of Sao Paulo (approval number CAAE 06191512.9.0000.5426).

Participants were excluded if they had experienced muscle injury in the past 6 months and/or were currently taking chronic or daily doses of anti-inflammatory medication or nutritional supplements. Participants who had a history of cardiovascular diseases were excluded from the study (McBrier et al., 2010). Participants were instructed to maintain their habitual diet throughout the study and to record their diet 1 day before performing the data collection and 3 days during the trials.

### Experimental design

A double blind, crossover with 2-week washout study design was conducted. Participants were assigned to one of two independent supplementation groups: Taurine + Chocolate milk (TAUchoc) and Placebo + Chocolate milk (CHOC). The study consisted of an 8-week supplementation period.

**Figure 1:**
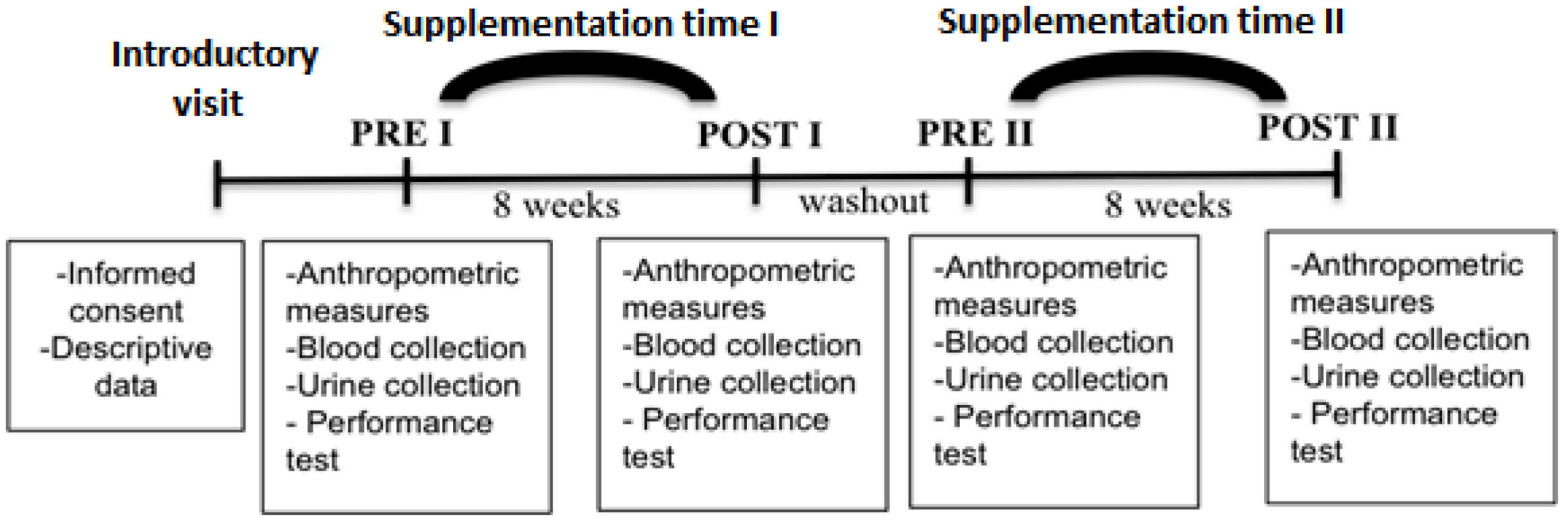
Schematic representation of the experimental trial. Subjects reported to the lab for an introductory visit, and then at PRE I the first treatment was started. After 8-weeks of treatment, all the measurements were repeated (POST I). A 2-week wash out was implemented between treatments, and then the protocol was repeated with the other treatment (PRE II and POST II).

Subjects were required to attend the laboratory eight times. On the first visit (PRE), the participants arrived at the laboratory in the morning after a 12 h overnight fast. Anthropometric measures including height, weight, and blood samples were taken and body fat percentage was determined by the deuterium method (Schoeller et al., 1980). The participants were asked to collect 24 h urine on the day before the data collection. After all procedures were completed, the first period of treatment was started. A maximal incremental running test was performed to determine aerobic capacity. Following the 8 weeks of supplementation with taurine or placebo, the subjects were invited to the lab to re-perform all evaluations (POST). A 2-week wash out was allowed and then the protocol was repeated with the other supplementation (PRE and POST second trial) (Figure 1).

After the baseline measurements, the subjects began the treatment with 3 grams of taurine (Aminoethylsulfonic Acid, 99% pure, Ajinomoto®) (Shao and Hathcock, 2008), or a placebo (starch), in capsules orally combined with low fat chocolate milk (CM) (400 ml) (Pepsico Co, Sao Paulo, SP) immediately after practice and again 2 hours post exercise, daily for 8 weeks. After a 2-week washout period, the protocol was repeated with the other treatment (taurine or placebo capsules). During the supplementation period, subjects were asked not to change their dietary habits and continue their triathlon training. The participants were required to record daily training, including data about hours and training zone, in order to calculate training load during each treatment. The training load results were reported in arbitrary units (au).

The low fat CM (Low Fat, Pepsico Chocolate milk, ready to drink, Sao Paulo, SP) contained 92 kcal, 16 grams of carbohydrate, 4 grams of protein and 1.2 grams of lipids per serving (200 ml). The CM was selected considering the optimal ratio of carbohydrate to protein (4:1), in order to favor glycogen resynthesis (Ivy et al., 2003; Kerksick and Leutholtz, 2005). In addition, CM presents a good taste and high acceptability by athletes (Karp et al., 2006; Pritchett et al., 2009).

### Taurine supplementation

The supplementation consisted of capsules containing 3 grams of pure taurine (*Aminoethylsulfonic Acid*, Ajinomoto®, São Paulo, SP) (Shao and Hathcock, 2008), or a placebo. The placebo consisted of a similar capsule containing starch. Subjects received three capsules daily (taurine or placebo) and 200 ml of low fat chocolate milk (CM) (LowFat Chocolate milk Pepsico, Sao Paulo, SP) (92 kcal, 16 g carbohydrate, 4 g protein, and 1.2 g fat) immediately after exercise and a further 200 ml of CM 1 h post exercise for a total of 60 days. After a 2-week washout period, the protocol was repeated with the other supplementation (taurine or placebo capsules). Taurine and placebo capsules were manipulated by the Department of Industrial Pharmacy of the School of Medicine of Ribeirão Preto, University of São Paulo.

### Dietetic assessment

Subjects were instructed to complete a 3-day food record and record dietary intake on the day before data collection and three non-consecutive days during each trial, in order to control for dietary intake. ESHA software (Esha Research Inc, Salem, OR) was used to examine kcal, carbohydrate, protein, and fat content.

### Measurements

#### Taurine assay

Plasma taurine was determined by high-performance liquid chromatography (Shimadzu, model LC 10AD). Taurine 99% was used as standard (Sigma-Aldrich, St. Louis, MO, USA) following the method of Deyl et al. (1986).

#### Oxidative stress markers

Blood was collected in 5 ml tubes containing separator and clot activating gel at PRE and POST supplementation after a 12-h fast. The samples were then stored in a freezer at −80°C until further analysis. The following oxidative stress markers indicative of lipid peroxidation were determined: reduced glutathione (GSH) according to the method of Sedlak and Lindsay (1968) and malondialdehyde (MDA) using the method proposed by Gerard-Monnier et al. (1998), with some adaptations. Blood vitamin E (total α-tocopherol) was determined by the method of α-tocopherol (Fabianek et al., 1968).

#### Protein metabolism

Twenty-four hour urine samples were collected PRE and POST each trial in order to quantify urinary excretion of nitrogen, creatinine and urea. Total nitrogen excretion was determined using 20 μL of 24-h diluted in 1,000 μl of distillated water using a chemiluminescence nitrogen analyzer according to the method proposed by Grimble et al. (1988). The nitrogen balance was calculated considering total nitrogen excretion and protein intake [NB = (Protein intake (g)/6.25) – Total nitrogen excretion (g) + 4 (g)] (Mendley and Majkowski, 2000). Creatinine and urea concentrations were determined by colorimetric reaction with a spectrophotometer using a Creatinine and Urea CEkit *(Labtestdiagnóstica*®).

#### Aerobic parameters

All subjects performed a maximal incremental running test with 1% gradient on a treadmill (Super ATL, Inbramed, Brazil) in a controlled environment. The test began at 8 km·h^−1^and speed was increased by 1 km·h^−1^ every 3 min until volitional exhaustion. Immediately after each stage, capillary blood samples from the ear lobe (25 μl) were assessed and blood lactate concentration was determined using a lactate analyzer (YSI 2300, Yellow Springs, Ohio, USA). A heart rate (HR) monitor (Polar, RS400, Finland) was used to measure heart rate and a 0–10 Foster's scale (Foster et al., 2001) to determine rate of perceived exertion (RPE) for each stage.

Lactate threshold intensity (LT) was determined using the D-max method, in which the points obtained through the speed, lactate concentration relationship were adjusted linearly and exponentially, the greatest distance between these two adjustments corresponded to anaerobic threshold intensity (Cheng et al., 1992). Maximum aerobic velocity (V_max_) was determined as the final completed stage during the incremental protocol.

### Statistical analysis

A *t*-test was used to compare percentage change (Δ% POST-PRE, TAUchoc vs. CHOC) between post and pre-supplementation for oxidative stress markers (MDA, GSH, and Vitamin E), taurine levels, and aerobic parameters between trials. Dietary assessment data from the two-supplementation protocols was compared with a *t*-test for independent samples. The paired *t*-test was used to compare changes within the trials. In cases of non-parametric distribution, the Wilcoxon test was used for comparisons within trials, and the Mann-Whitney test for comparisons between trials. Statistical Package for the Social Sciences (SPSS) for Windows software, version 15.0, was used for all statistical analyses. All data are reported as means ± standard deviation. Statistical significance was set at *p* < 0.05 for all analyses.

## Results

Participant characteristics are reported as means ± SD for each participant as follows: age (years): 30.9 ± 1.3, height (cm): 179.0 ± 0.01, weight (kg): 77.45 ± 2.4, body fat mass: 15% ± 1.5, and body lean mass: 85% ± 1.4. According to the dietary analysis, there were no significant differences in kcal or macronutrient intake (carbohydrate, protein, and fat). The TAUchoc group consumed, on average, 2,243.7 ± 770.7 kcal, 4.29 ± 1.35 g/kg of body weight (BW) of carbohydrate, 1.78 ± 0.57 g/kg BW of protein, and 0.89 ± 0.28 g/kg BW of lipids, while the CHOC group consumed, on average, 2,122.6 ± 702.4 kcal, 4.17 ± 1.31 g/kg BW of carbohydrate, 1.61 ± 0.51 g/kg BW of protein and 0.91 ± 0.23 g/kg BW of lipids.

With regard to plasma concentration of taurine in the TAUchoc and CHOC groups there was no difference in baseline concentrations (*p* = 0.68). A significant increase in taurine plasma levels was observed with TAUchoc supplementation (PRE 201.32 ± 29.03 μmol/L and POST 234.36 ± 35.51, *p* = 0.01), but not with CHOC supplementation PRE 208.51 ± 38.04 μmol/L and POST 191.71 ± 24.96 μmol/L, (*p* = 0.21) after 8 weeks of supplementation.

No significant changes were found in GSH or vitamin E levels between trials (TAUchoc and CHOC) and comparing PRE to POST supplementation (Table 1). However, a significant decrease (−21%) was observed in MDA levels after TAUchoc supplementation (*p* = 0.03), suggesting that taurine prevented lipid peroxidation.

**Table 1:** Oxidative stress and protein metabolism marker levels PRE and POST supplementation (TAUchoc and CHOC).

**Measurements**	**TAUchoc**			**CHOC**		
	**PRE**	**POST**	**Δ% (POST-PRE)**	**PRE**	**POST**	**Δ% (POST-PRE)**
GSH (mmol/L)	0.72 ± 0.08	0.83 ± 0.08	16.94	0.69 ± 0.08	0.81 ± 0.06	24.24
Vitamin E (μmol/L)	33.99 ± 2.52	35.95 ± 2.80	6.54	31.48 ± 2.12	33.77 ± 3.64	6.54
MDA (μMl)	3.62 ± 0.64	2.86 ± 0.05	−21^*^	4.38 ± 0.60	4.30 ± 0.64	−0.46
N ur (g/day)	26.91 ± 3	16.70 ± 1.9^**^	−37.9^**^	24.2 ± 2.7	20.20 ± 1.90	−16.5
Urea (g/24 h)	37.53 ± 3.80	29.34 ± 2.5	−21.9	32.04 ± 4.85	37.91 ± 6.87	18.32
Creatinine (mg/Kg/24 h)	28.14 ± 2.68	22.06 ± 2.97	−21.06	23.05 ± 2.26	26.73 ± 1.98	15.9
NB (g/24h)	−4.84±−1.4	6.20 ± 1.79^#^	228.1	1.24 ± 0.36	4.91 ± 1.42	295.9

GSH, reduced glutathione; MDA, malondialdehyde; N ur, 24 h nitrogen excretion; NB, nitrogen balance. TAUchoc, taurine associated with chocolate milk supplementation; CHOC, chocolate milk and placebo supplementation; Δ% (POST-PRE), percentage change post to pre supplementation; PRE, pre supplementation; POST, post supplementation.

* Significant decrease was observed in MDA levels post TAUchoc treatment.

** Significant decrease in relation to CHOC group (pared t test, p ≤ 0.05).

# *significant increase was observed in NB post TAUchoc treatment. Data reported as means ± SD. n = 10*.

A significant reduction in 24 h urinary nitrogen excretion was observed following the TAUchoc treatment (*p* = 0.03) and the percentage variation was higher post TAUchoc treatment (−37.9%) when compared to CHOC treatment (−16.5%) (Table 1) (*p* = 0.03). Nitrogen balance was calculated considering protein intake and total urinary nitrogen excretion. Although no significant differences were found in protein intake, a significant change was observed in nitrogen balance following TAUchoc treatment (*p* = 0.01), changing from negative (−4.84 ± 1.4 g/24 h) to positive (6.20 ± 1.79 g/24 h).

**Figure 2:**
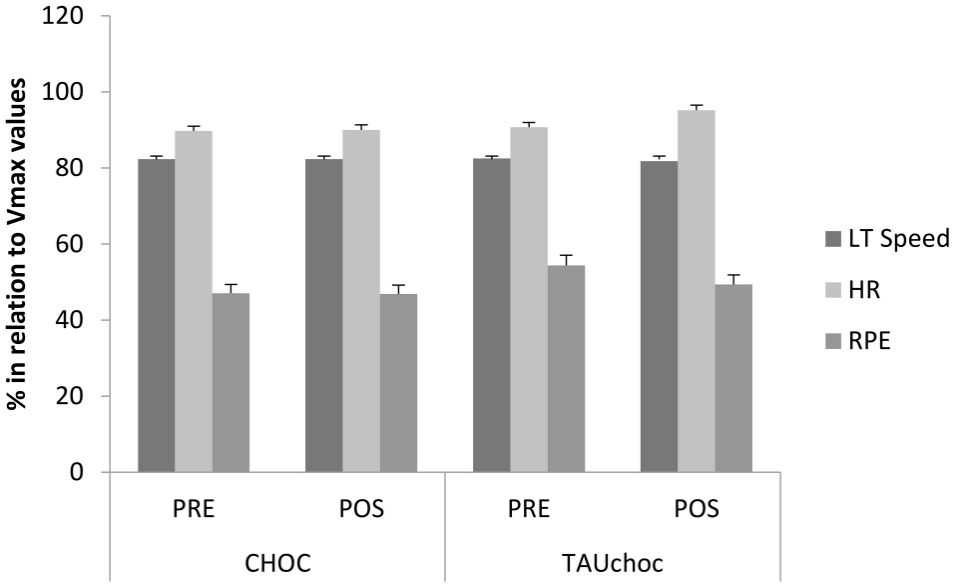
Relative percentage change calculated considering the variable values at LT compared to values at V_max_ (*n* = 10). This graph shows that groups were similar before starting the supplementation and no changes were observed when analyzing pre and post values at LT compared to values at V_max._ CHOC, chocolate milk and placebo treatment. TAUchoc, Taurine and chocolate milk treatment. LT Speed, Speed at lactate threshold. HR: heart rate. RPE, rate of perceived exertion. V_max_, maximum aerobic velocity. No changes were observed post TAUchoc and CHOC supplementation.

Regarding the aerobic parameters, which were obtained in the last phase of the maximal incremental running test, no significant changes were observed for V_max_(TAUchoc PRE 13 ± 1.4 km/h and POST 13.22 ± 1.34 km/h; CHOC PRE 13.11 ± 2.34 km/h and POST 13.11 ± 2.72 km/h). Heat rate (HR) values were TAUchoc PRE 181.89 ± 24.18 bpm and POST 168.89 ± 46.56 bpm; CHOC PRE 181.56 ± 2.14 bpm and POST 179.78 ± 3.4 bpm, and the RPE values were TAUchoc PRE 8.33 ± 2.4 AU and POST 9.1 ± 2.1 AU; CHOC PRE 8.11 ± 4.94 AU and POST 8.78 ± 2.78 AU. Figure 2 displays a relative percentage calculated considering the variable values at LT compared to values at V_max_; it was found that the groups were similar before starting the supplementation and there were no significant changes in aerobic parameters post supplementation for either group (TAUchoc and CHOC).

## Discussion

The main purpose of this study was to examine the additive effects of TAUchoc vs. CHOC on indices of oxidative stress, protein catabolism, and aerobic capacity in triathletes. Exercise performance was assessed using an incremental running test. Our results suggest that supplementation with taurine or a placebo associated with low fat chocolate milk for an 8-week period did not improve aerobic performance, however the taurine with chocolate milk (TAUchoc) provided benefits to oxidative stress markers and increased taurine plasma levels, demonstrating that the supplementation protocol was effective.

Regarding oxidative stress, moderate training appears to benefit health and oxidative stress since exercise stimulus is necessary to promote up-regulation in endogenous antioxidant defenses (Pingitore et al., 2015), however strenuous aerobic exercise such as triathlon, can induce reactive oxygen species overproduction and may cause oxidative damage if the endogenous defense is not effective, thereby compromising athletic performance (Hawley, 2009). Therefore, utilization of antioxidant compounds post exercise aim to prevent oxidative stress and possibly enhance performance (Kim and Lee, 2014; Pingitore et al., 2015).

Also, taurine has been considered in the scientific literature a nutraceutical compound due to its various beneficial effects on human health (Xu et al., 2008) such as the treatment of fatigue and myotonia (Trip et al., 2006), visual protection in diabetics (Yu et al., 2008), immunocompetence improvement (Grimble, 2006), anti-neurotoxic and anti-inflammatory effects, and tumor cell proliferation inhibition (Schuller-Levis and Park, 2003; Grimble, 2006; Klusa et al., 2006; Marcinkiewicz et al., 2006). Therefore, taurine supplementation can benefit athletes metabolism by the modulation of the inflammatory process and oxidative stress.

The results of the present study indicated that taurine supplementation was effective in decreasing lipid peroxidation due to a 21% reduction in MDA levels after TAUchoc treatment, however no changes were found in antioxidant markers (GSH and Vitamin E). Even though the results were not significant, it is important to note that the average levels of GSH and α-tocopherol post treatment were higher in the TAUchoc supplementation than the CHOC supplementation. These results suggest that TAUchoc supplementation prevented the utilization of GSH and vitamin E against free radicals and improved the activities of the antioxidant defense system.

Similar results were found by Zhang et al. (2004) who evaluated the effects of 7-day taurine supplementation (6 g/day) on oxidative stress induced by exercise in cyclists, and concluded that taurine decreased levels of thiobarbituric acid reactive substances (TBARS), which are also indicators of lipid peroxidation. Silva et al. (2011) investigated the effects of a 15-day taurine supplementation (300 mg/kg) or saline on oxidative stress biomarkers after a 90-min downhill run session in rats and concluded that taurine affected skeletal muscle contraction by decreasing oxidative stress (superoxide radical production, creatine kinase, lipid peroxidation, and carbonylation levels), however no changes were found in the antioxidant enzyme activity after the exercise protocol (Silva et al., 2011).

It was expected that taurine may improve athletes performance. According to Dutka et al. (2014), taurine can improve muscle function and blood lactate levels by the following mechanisms: (1) an interaction between the skeletal muscle membrane and taurine; (2) an increase of calcium release to contractile filaments of skeletal muscle, which enhances force production; (3) an increase of mitochondrial buffering. In addition, Ward et al. (1999) suggested that the intensity and speed performed during exercise are strongly correlated with high taurine levels, possibly indicating its release of muscle fibers (Ward et al., 1999).

Also, some studies suggested that the high levels of taurine may reflect changes in the electrophysical properties of skeletal muscle membrane or in blood osmolarity due to its co-release with water to maintain plasma volume and calcium homeostasis (Ward et al., 1999, 2016; Spriet and Whitfield, 2015). In addition, taurine is related to lactic acid buffering in brain and skeletal muscle cells in rats (Nakada et al., 1991). However, the blood lactate levels post exercise remained unaltered after taurine supplementation in athletes (Lee et al., 2003; Rutherford et al., 2010).

In addition, Pierno et al. (2012) showed an important action of taurine on muscular dysfunction, especially on the skeletal muscle disuse-induced impairment, which is related to the reduction of taurine content in postural muscles. The authors verified that taurine supplementation restored the expression of an atrophy-related gene (i.e., Muscle RING-Finger Protein 1/MuRF-1) in rats, suggesting a beneficial role of taurine in the prevention of disuse-induced skeletal muscle atrophy.

However, in the present study improvements in performance were not observed, since that no significant changes were detected in aerobic parameters post TAUchoc and CHOC supplementation. It is important to highlight that, even though not significant, a short increase in V_max_ (0.15 Km/h) POST compared to PRE TAUchoc and a decrease in HR with post TAUchoc were observed in the maximal incremental running test performed. These results indicate that taurine may have improved cardiorespiratory response during exercise. Therefore, TAUchoc treatment may offer some practical performance benefits, especially when observing the results of the last Ironman Brazil race, which took place in Florianópolis city, Brazil, where the time difference to finish the race between the first and second places was 18 min and 6 s, and between the second and third places was 42 s (Ironman Brasil, 2016). Therefore, small increases in performance, mainly the speed developed during a race, may result in success in the race, which suggests that a triathlete who runs 0.15 km/h faster than another athlete post TAUchoc treatment may win a race even if the difference in the speed performed along the race between de winner and the second place athlete is not statistically significant.

Other researchers investigated a cycling time trial performance (5 kJ of work/kg body mass as fast as possible) with taurine supplementation and did not demonstrate improvement in performance (11). Controversially, Balshaw et al. (2013) investigated the effect of acute ingestion of 1 g of taurine on maximal 3 km time trial (3KTT) performance in trained middle-distance runners, and found that the ingestion of taurine improved 3KTT performance by 1.7%.

Concerning time of administration, according to Ghandforoush-Sattari et al. (2010), maximum plasma taurine concentration may be reached 1.5 ± 0.6 h post administration. Therefore, the TAUchoc treatment was expected to increase the availability and utilization of nutrients post exercise and consequently stimulate glycogen synthesis and promote protein synthesis. In the present study, the results related to urinary protein metabolism showed that TAUchoc supplementation induced lower urinary nitrogen excretion and positive nitrogen-balance. The nitrogen-balance demonstrates how much nitrogen is coming into the body and how much is being excreted. A positive nitrogen balance indicates that more nitrogen is being retained than excreted, which suggests that muscle is being gained (Benardot, 2006).

Although other authors have shown changes in urea levels post intense exercise training (Benardot, 2006), the present study did not find changes in urea levels after TAUchoc and CHOC and 8 weeks of triathlon practice. Conversely, with different sports modalities, no significant changes in urinary urea levels were found after a periodized training in a study with soccer players (Silva et al., 2006) and cyclists (Halson et al., 2002). According to Halson et al. (2002) creatinine and urea can be used as markers of muscle damage, changes in lean body mass, and dehydration. Since no changes were found in urea and creatinine levels, and nitrogen balance was positive after TAUchoc treatment, the results of the present study suggest that taurine can contribute to preservation of muscle mass and may favor muscle recovery by ensuring adequate nutrient supply to muscle cells post exercise.

Although no significant treatment effects were observed in the aerobic parameters, our results indicate that there may be potential performance benefits attributed to taurine supplementation. From a practical perspective, an increase of 1% in the speed was observed in the LT of the subjects in the TAUchoc group when comparing with the test speed developed before treatment (PRE). Therefore, the association of taurine and chocolate milk as a post exercise recovery beverage may offer practical performance benefits for an athlete during a competition.

## Conclusion

The results of the present study indicated that taurine supplementation did not improve aerobic parameters, but was effective in increasing taurine plasma levels and decreasing oxidative stress markers, which suggests that taurine may prevent oxidative stress in triathletes. Additional studies are warranted to examine the potential advantages of taurine supplementation on indices of exercise recovery and muscle damage, since some evidence of performance improvement was observed in the present study.

## Author contributions

FGDC, BSMG, PCS, and ECF design of the work; acquisition, analysis and interpretation of data for the work; paper writing; KeP, KaP, EF, MP, JSM, and ECF analysis and interpretation of data for the work, critical revising for important intellectual content; and final approval of the version to be published.

### Conflict of interest statement

The authors declare that the research was conducted in the absence of any commercial or financial relationships that could be construed as a potential conflict of interest.
